# Immunity, the God-given weapon against infection

**DOI:** 10.1093/nsr/nwv035

**Published:** 2015-06-30

**Authors:** Xuetao Cao

**Affiliations:** Xuetao Cao Professor & President, Chinese Academy of Medical SciencesGuest Editor of Special Topic & Editorial Board Member of NSR

The development of human civilization is a history of struggling with diseases. In the past, people have suffered a lot from the epidemic of infection diseases such as cholera and smallpox. For a long time, people were helpless and had no useful tools to counteract these diseases. It is not until recent several hundred years that the human beings gradually seize the initiative in this battle. People gradually find out the prime culprit for infection and learn to know about infectious diseases. More importantly, the utilization of vaccine is a fundamental sign of humans’ counterattack to infection diseases.

As a host protection mechanism, the immune system can discriminate non-self components, and then initiate innate (non-specific) and adaptive (specific) immune response to eliminate the non-self components such as invading pathogens. The past few decades witness a fast development in the research of immunology. The discovery of pattern recognition receptors, such as Toll-like receptors, RIG-I-like receptors and cytosolic DNA sensor cGAS, has greatly enriched our understanding on how the body senses invading pathogens and primes innate immune responses. Besides, new subsets of immune cells have been identified in recent years. For example, regulatory T cells, Th17 and innate lymphoid cells are found to have broad roles in immune responses against infections with bacteria and viruses. Based on the advances in immunological research, novel drugs and therapeutic approaches are applied for the control of infectious diseases and acquired favorable therapeutic effects. Although many progresses have been achieved, we have to face new challenges. On the one hand, pathogens are consistently evolving to escape the surveillance of the immune system. The drastic antigenic changes in influenza viruses have exceeded the development of efficient vaccines and caused lethal threats to vulnerable people. The emergence of multiple resistant bacteria has led doctors to the edge of powerlessness. On the other hand, the ever growing population and its mobility makes the wide spread of pathogens much easier. More and more infection diseases have become public health problems. It is still clearly remembered the epidemic of severe acute respiratory syndrome caused by coronavirus in 2003 in China. Recently, the widespread outbreak of Ebola virus in West Africa has again alerted us. These facts show the urgency for the in-depth investigation of the immunologic mechanism during infection and the development of new drugs and vaccines.

To introduce recent progresses in the field of infection and immunity, we dedicate this issue of *National Science Review* to the frontiers of anti-infectious immunity. We start with a Research Highlight by Zhubo Chen on a recent study by Jing Wang *et al.* for revealing a new mechanism of *Mycobacterium tuberculosis* to subvert innate immunity. We then present a Perspective by Xingguang Liu on the molecular regulation of type I interferon production in MAVS- and STING-mediated antiviral innate responses, followed by a Perspective by Tao Dong on cytotoxic T lymphocytes-mediated immunity in influenza virus infection and another Perspective by Xiaofeng Qin *et al.* that introduces promising therapeutic targets for preventing Ebola viral infection identified though systematic research approaches. In the subsequent review article by Jianwei Wang *et al.*, they intensively discuss the present understanding of hand, foot and mouth diseases. We also present a review by Jin Zhong *et al.* that shares their views on the rationale and strategy to develop an effective hepatitis C virus vaccine, followed by another review article by Zhenghong Yuan *et al.* on the research progresses and therapeutic developments in hepatitis B virus infection. Finally, an interview with Lanjuan Li and Yi Shi provides their perspectives on the basic research and clinical treatment of infectious diseases.

As the guest editor of this special topic in infection and immunity, I would like to express my thanks to all of the authors, reviewers and the editorial staff for their dedication to this special topic. The battle against infection will accompany with human for a long time. We are pleased that immunological research is being supported with more and more funding from the government, and we should be optimistic that the rapid advances in immunological research will help us grip this God-given weapon to defeat infection diseases.

